# Brain Activation Induced by Myopic and Hyperopic Defocus From Spectacles

**DOI:** 10.3389/fnhum.2021.711713

**Published:** 2021-09-14

**Authors:** Meng-Tian Kang, Bo Wang, An-Ran Ran, Jiahe Gan, Jialing Du, Mayinuer Yusufu, Xintong Liang, Shi-Ming Li, Ningli Wang

**Affiliations:** ^1^Beijing Ophthalmology and Visual Science Key Lab, Beijing Tongren Eye Center, Beijing Tongren Hospital, Capital Medical University, Beijing, China; ^2^Beijing Institute of Ophthalmology, Beijing Tongren Eye Center, Beijing Tongren Hospital, Capital Medical University, Beijing, China; ^3^State Key Laboratory of Brain and Cognitive Science, Institute of Biophysics, Chinese Academy of Sciences, Beijing, China; ^4^Department of Ophthalmology and Visual Sciences, The Chinese University of Hong Kong, Hong Kong, China

**Keywords:** fMRI, myopia, defocus, neural change, arterial spin labeling

## Abstract

**Purpose:** To assess neural changes in perceptual effects induced by myopic defocus and hyperopic defocus stimuli in ametropic and emmetropic subjects using functional magnetic resonance imaging (fMRI).

**Methods:** This study included 41 subjects with a mean age of 26.0 ± 2.9 years. The mean spherical equivalence refraction was −0.54 ± 0.51D in the emmetropic group and −3.57 ± 2.27D in the ametropic group. The subjects were instructed to view through full refractive correction, with values of +2.00D to induce myopic defocus state and −2.00D to induce hyperopic defocus state. This was carried over in three random sessions. Arterial spin labeling (ASL) perfusion was measured using fMRI to obtain quantified regional cerebral blood flow (rCBF). Behavioral tests including distant visual acuity (VA) and contrast sensitivity (CS), were measured every 5 min for 30 min.

**Results:** Myopic defocus induced significantly greater rCBF increase in four cerebral regions compared with full correction: right precentral gyrus, right superior temporal gyrus, left inferior parietal lobule, and left middle temporal gyrus (*P* < 0.001). The differences were less significant in low myopes than emmetropes. In the hyperopic defocus session, the increased responses of rCBF were only observed in the right and left precentral gyrus. Myopic defocused VA and CS improved significantly within 5 min and reached a plateau shortly after.

**Conclusion:** This study revealed that myopic defocus stimuli can significantly increase blood perfusion in visual attention-related cerebral regions, which suggests a potential direction for future investigation on the relationship between retinal defocus and its neural consequences.

## Introduction

Increasing evidence from a diverse range of animal studies indicates that ocular growth is strongly guided by visual error signals, specifically the optical defocus (Wildsoet, 1997; Wallman and Winawer, 2004). When myopic defocus induced by the lens (image-focused anterior to the retina) is imposed on the eyes of the animals, the eye rapidly compensates and becomes hyperopic by altering the depth of the vitreous chamber, increasing choroidal thickness, and slightly decreasing in ocular length (Wallman et al., 1995; Wildsoet and Wallman, 1995; Hung et al., 2000; Zhu et al., 2013). These responses function to reduce retinal blur and, thus, are generally accepted as a foundation for emmetropization, an optically guided process essential to the normal growth of the eye. In the study of Wildsoet and Wallman, they found that when interfering with neural communication between the retina and the brain of a chick by optic nerve section, hyperopic defocus induced myopia was impaired (Wildsoet and Wallman, 1995). However, little is known about what cues does the retina use to discern the defocus signal.

A perceptual mechanism has been shown to contribute to the regulation of image defocus. Blur adaption experiment suggested that myopic defocus could lead to visual function improvement as prolonged exposure to optical defocus would increase the ability to detect and recognize letters (Mon-Williams et al., 1998). In addition, blur adaptation improves the visual quality by adjusting the spatial frequency signal pathway through the neural adaptation process to maintain the spatial structure of the image (Webster et al., 2002). The study of Dillingham found that cutting the optic nerve nucleus of the isthmus (the connection between the central nervous system [CNS] and the retina) induced initial, transient hyperopia in the contralateral eye. This finding implicates ipsilaterally projecting centrifugal neurons in the regulation of emmetropization mechanisms, a process that may take over *via* nitric oxide/dopaminergic pathways. It further proves that light-dependent ocular development is closely related to the CNS (Dillingham et al., 2016).

The aim of the current study is to investigate two aspects of CNS and defocus stimuli: (1) the short-term effect of myopic and hyperopic defocus on cerebral blood flow (CBF), and (2) whether the response varies along with refractive status. Studying the effect of defocus signal on human brain function can facilitate our understanding of the changes in the human advanced nervous system caused by the optical correction.

## Methods

### Subjects

A total of 41 subjects participated in this study. The inclusion criteria were as follows: (1) age ranged from 18 to 35 years, with either emmetropia or ametropia, (2) no more than 1.00D astigmatism and <1.50D anisometropia binocularly, and (3) had a normal or corrected-to-normal vision of logarithm of the minimum angle of resolution (logMAR) visual acuity (VA) 0.00 (Snellen 6/6 or 20/20) or better. The exclusion criteria were as follows: (1) any history of amblyopia, ocular pathologies, or other ocular anomalies (e.g., surgery, trauma) that might have influenced the measurement, and (2) any mental or nervous system disease, cardiovascular diseases, or other symptoms unsuitable for MRI scanning. Emmetrope was defined as having spherical equivalent refraction (SER) of +0.75D to −0.75D inclusive. Myope was defined as having a negative SER of at least −0.75D and was classified into three levels as low, moderate, and high myope (−3 to −0.75D, −6 to −3D, and ≤-6D, respectively). All procedures in this research adhered to the tenets of the Declaration of Helsinki and the experimental procedure was approved by the Institutional Review Board of Beijing MRI Center for Brain Research (BMCBR).

### Ocular Examinations

The researchers performed ocular examinations for the subjects after explaining the nature and procedures of the study to them and obtaining their signed consent. Ocular examinations included logMAR VA, dominant eye test, manifest and cycloplegic refraction (Auto Ref/Keratometer WAM-5500, Grand Seiko, Japan). Tropicamide eye drops were administered two times for every 15 min during the examinations. In addition, the prescription of glasses, head circumference, height, and weight of the subjects were measured.

### Behavior Test

The logMAR VA and contrast sensitivity (CS) were both measured monocularly at 5 min intervals over 30 min. During the course, all the emmetropes and full-corrected myopic subjects wore a non-magnetic trial frame with a convex plastic lens of +2D to induce the myopic defocus state. The subjects maintained a distance fixation of 4 m throughout the trial period. All the VA measurements were taken from the right eye, whereas the left eye was fully occluded.

### Magnetic Resonance Imaging

Magnetic resonance imaging was performed on a Siemens 3T Prisma scanner (Erlangen, Germany) with a 20-channel receive head coil in BMCBR. During MRI scanning, subjects were asked to lie down still in the scanner, and the head movement was minimized by two pieces of foam surrounding the head of the subjects.

High-resolution (1 × 1 × 1 mm^3^) structural image was acquired with a 3D magnetization-prepared rapid gradient-echo (MP-RAGE) sequence for T1 weighted (TR = 2530 ms, TE = 3.25 ms, flip angle = 9°, FOV = 256 × 256 mm, matrix = 256 × 256, 176 sagittal slices with 1 mm thickness).

**Figure 1:**
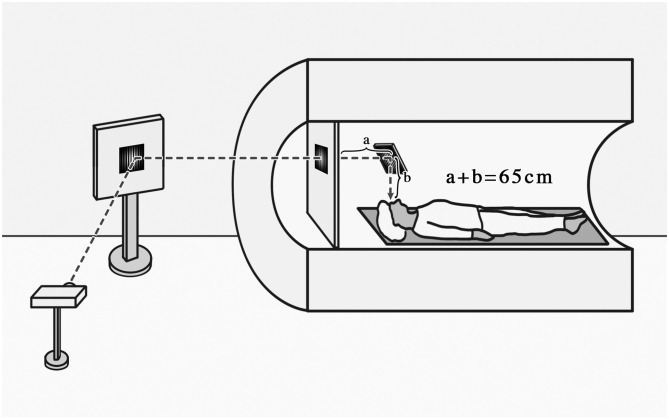
Schematic diagram of the subjects receiving visual tasks.

Arterial spin labeling (ASL) perfusion MRI, which uses magnetically labeled arterial water as an endogenous tracer to obtain quantified CBF maps, was performed with 3D gradient and spin-echo (GRASE) pseudo-continuous arterial spin labeling (pCASL) sequence (TR = 4,000 ms, TE = 17.96 ms, FOV = 192 × 192 mm, voxel size = 3 × 3 mm, 24 slices acquired in ascending order, slice thickness = 5 mm with 1 mm gap between slices, labeling duration = 1,650 ms, post labeling delay = 1,200 ms, number of controls/labels = 30 pairs). The stimuli were generated by a computer and were back-projected on a screen located inside the MRI bore in front of the head of the patient. Subjects viewed the screen at a total path length of 65 cm through a mirror situated above their eyes (Figure 1).

### Apparatus and Stimuli

Custom algorithms written in MATLAB were used in all experiments to obtain stimulated images blurred with defocus. All the images were with a size of 613 × 613 pixels and presented as vertical black and white grating whose blur was manipulated (Sawides et al., 2010). The blur of the grating was intermediate between that of a square grating and a sine grating. This was achieved by replacing each of the sharp edges of a square grating with half cosine wave luminance profiles.

### Procedure

Each subject attended three measurement sessions, each conducted in a randomized order. The CBF images were obtained before and at 15 min intervals, it was observed during 7 min whether the spectacle induced +2D spherical myopic defocus, −2D spherical hyperopic defocus, or clear vision. All the emmetropes and full-corrected myopic subjects wore a non-magnetic trial frame with a plastic lens of +2D to induce a myopic defocus state or a plastic lens of −2D to induce a hyperopic defocus state. The emmetropes wore plain glasses, and the myopic subjects wore glasses that provide the full correction to induce clear focus. During each experimental session, the subjects were asked to have a 7-min binocular distant viewing tasking of watching a grating image with 613 × 613 pixels projected on a translucent screen (subtending visual field of 60° at a viewing distance of 65 cm). The image was projected on the screen through a tilted mirror mounted on the head coil to achieve optimal viewing distance. The experiments were performed in a room with an illumination of 10 lux. Subjects were unknown to the effect of the stimulation condition.

### Data Processing

The first two volumes of each functional run were discarded to allow for magnetization equilibration. Data were analyzed using statistical parametric mapping (SPM8) (Wellcome Trust Centre for Neuroimaging, University College London, UK) under MATLAB. Other images were realigned and resliced to correct for head motion with a mean volume created. The subject whose head motion exceeded 3 mm or rotation exceeded 3° during scanning were excluded. All realigned echo-planar images (EPI) were subsequently smoothed using an 8 mm full-width at half-maximum (FWHM) isotropic Gaussian kernel in SPM8. Images of CBF were then reconstructed from preprocessed EPI images, and the acquired CBF images were averaged to mean image for each condition (1 resting and 3 visual tasks). The mean CBF images were finally normalized to standard Montreal Neurological Institute (MNI) space, and the voxels were resampled to isotropic 2 × 2 × 2 mm^3^.

The normalized mean CBF maps of defocus and clear focus were modeled in a paired *t*-test factorial design with SPM8. The brain areas showing a significant difference in the CBF between two visual tasks were filtered at threshold *P* < 0.001 (uncorrected, cluster size ≥30) as a region of interest (ROI).

The value of the regional CBF (rCBF) of each ROI over the threshold was extracted from the normalized mean CBF images and then normalized with the global mean of the whole volume. A paired *t*-test of the corrected rCBF values was conducted in SPSS 20 to reveal the significant difference between defocus signal and clear focus.

The individual who processed the data was masked to the stimulus condition.

## Results

After screening through the ophthalmic examination, 41 subjects met the inclusion criteria and completed the fMRI examination. According to their cycloplegic SER, the 41 subjects were grouped into emmetropes, low myopes, moderate myopes, and high myopes. Mean SER was −3.64 ± 2.35D in right eyes and −3.57 ± 2.27D in left eyes. There was no significant difference in mean age, gender, height, and weight among groups (*P* > 0.05, Table 1).

**Table 1:** Characteristics of the subjects.

	**All**	**Emmetropes**	**Low myopes**	**Moderate myopes**	**High myopes**
*N*	41	9	6	16	10
Age, mean (year)	26.0 ± 2.9	27.0 ± 4.4	25.5 ± 1.8	25.6 ± 2.3	25.9 ± 2.8
Gender (male/female)	15/26	6/3	2/4	3/13	4/6
Dominant eye (right/left)	22/19	4/5	3/3	9/7	6/4
Handedness (right/left)	40/1	8/1	6/0	16/0	10/0
Right eye SER, mean (D)	−3.64 ± 2.35	−0.53 ± 0.33	−2.22 ± 0.63	−4.02 ± 0.91	−6.67 ± 1.20
Left eye SER, mean (D)	−3.57 ± 2.27	−0.55 ± 0.51	−2.26 ± 0.40	−3.92 ± 0.99	−6.53 ± 0.84
Height, mean (cm)	168.2 ± 7.8	171.0 ± 7.6	168.3 ± 14.0	165.6 ± 5.2	169.9 ± 6.4
Weight, mean (kg)	61.7 ± 12.9	67.6 ± 13.8	61.8 ± 14.8	57.9 ± 11.1	62.4 ± 13.5

*SER, spherical equivalent refraction; D, diopter*.

**Figure 2:**
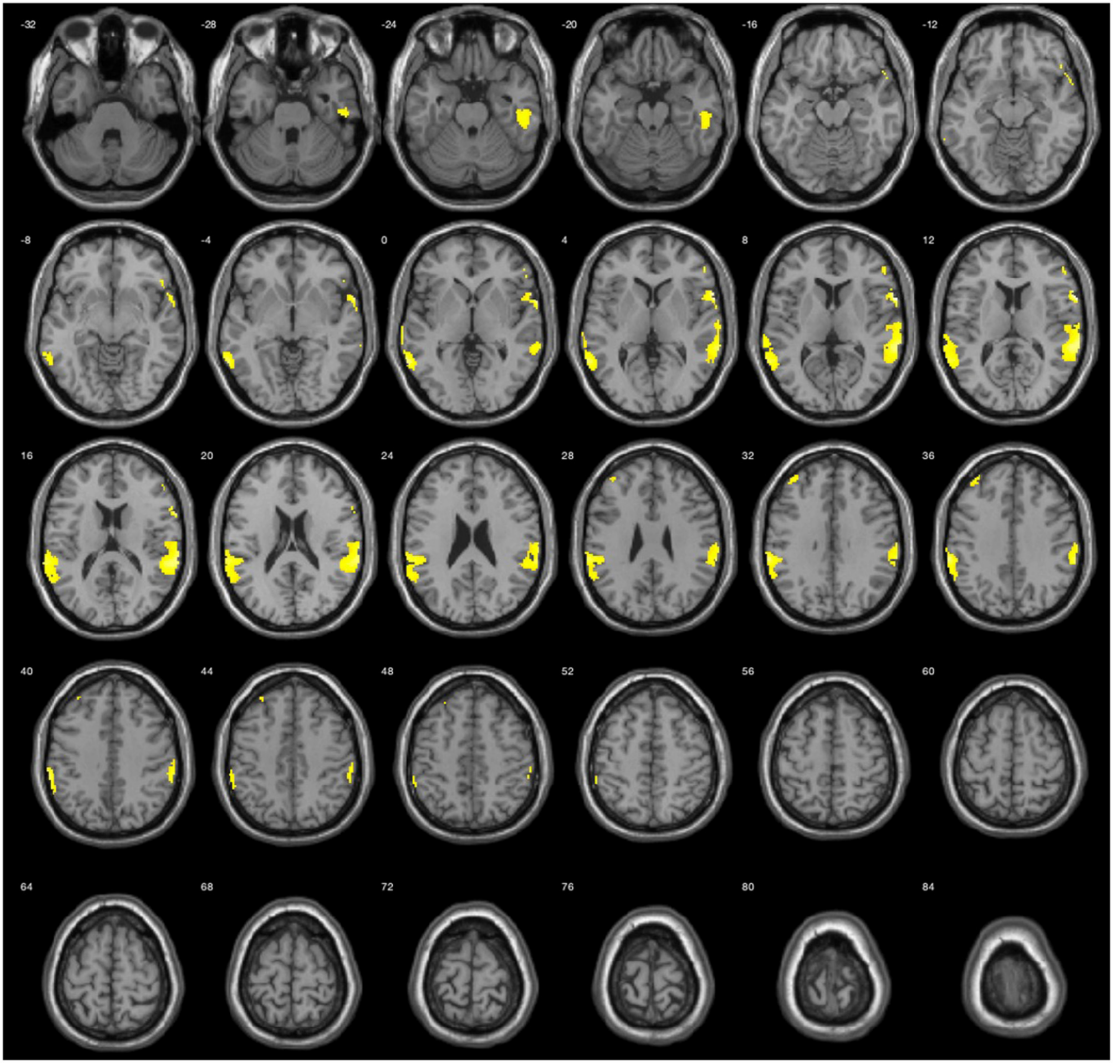
Average activation maps resulting from group analysis, showing the increase of neural activation in myopic defocus relative to clear focus state. Color scale indicates score significance level.

**Figure 3:**
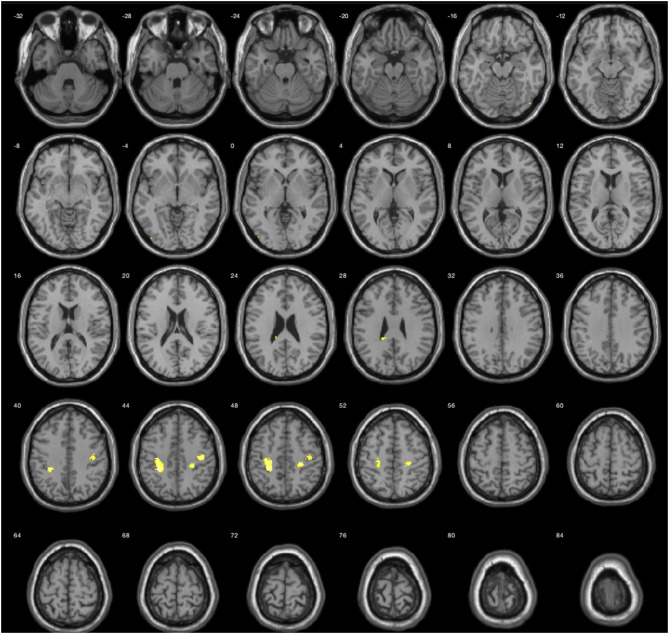
Average activation maps resulting from group analysis, showing the increase of neural activation in hyperopic defocus relative to clear focus state. Color scale indicates score significance level.

The results during the myopic defocus session showed increased CBF in several cerebral regions compared with clear focus stimulation (Figure 2, *P* < 0.001, cluster size ≥30, and Table 2). The rCBF of myopic defocus stimulation was significantly higher than clear focus in four ROIs: right precentral gyrus, right superior temporal gyrus, left inferior parietal lobule, and left middle temporal gyrus. The results during the hyperopic defocus session showed that the rCBF of hyperopic defocus stimulation was significantly higher than clear focus in the right precentral gyrus and left precentral gyrus (Figure 3, *P* < 0.001, cluster size ≥30, and Table 2).

**Table 2:** The active loci of myopic defocus and hyperopic defocus of increased regional cerebral blood flow (rCBF) response compared with clear defocus.

	**Activation loci**	**Brodmann area**	**Z**	**MNI coordinates** **x, y, z (mm)**		
Myopic defocus vs. clear focus	(1) Right precentral gyrus	44	4.59	66	10	8
	(2) Right superior temporal gyrus	22	4.06	66	−36	14
	(3) Left inferior parietal lobule	40	4.06	−64	−34	36
	(4) Left middle temporal gyrus	21	3.97	−62	−54	0
Hyperopic defocus vs. clear focus	(1) Right cerebrum, frontal lobe, precentral gyrus	4	4.98	26	−28	50
	(2) Right cerebrum, frontal lobe, precentral gyrus	4	4.51	44	−18	36
	(3) Left cerebrum, frontal lobe, precentral gyrus	40	4.93	−28	−36	44

**Figure 4:**
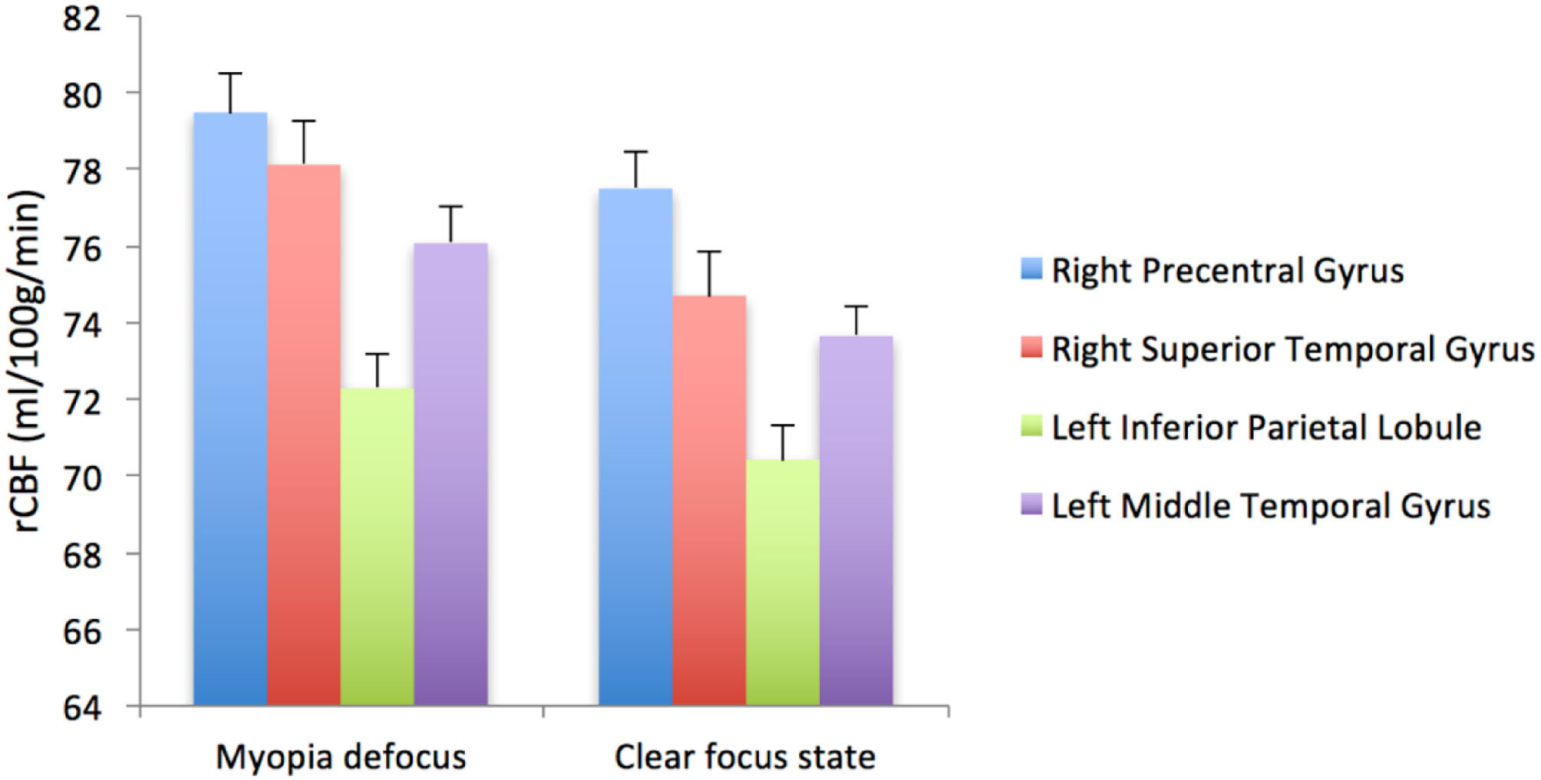
The rCBF signal intensity (ml/100 g/min) change in four ROI averaged over 41 subjects (error bars represent SEM).

When the brain received myopic defocus signal and the clear focus signal, the average elevation of rCBF was 78.12 ± 1.18 ml/100 g/min and 74.70 ± 1.26 ml/100 g/min respectively in the right precentral gyrus, 76.07 ± 0.78 ml/100 g/min and 73.67 ± 0.72 ml/100 g/min in the right superior temporal gyrus, 72.29 ± 0.90 ml/100 g/min and 70.42 ± 0.84 ml/100 g/min in the left inferior parietal lobule, and 79.48 ± 0.95 ml/100 g/min and 77.50 ± 0.89 ml/100 g/min in the left middle temporal gyrus. The differences between groups were statistically significant (*P* < 0.05) (Figure 4).

**Figure 5:**
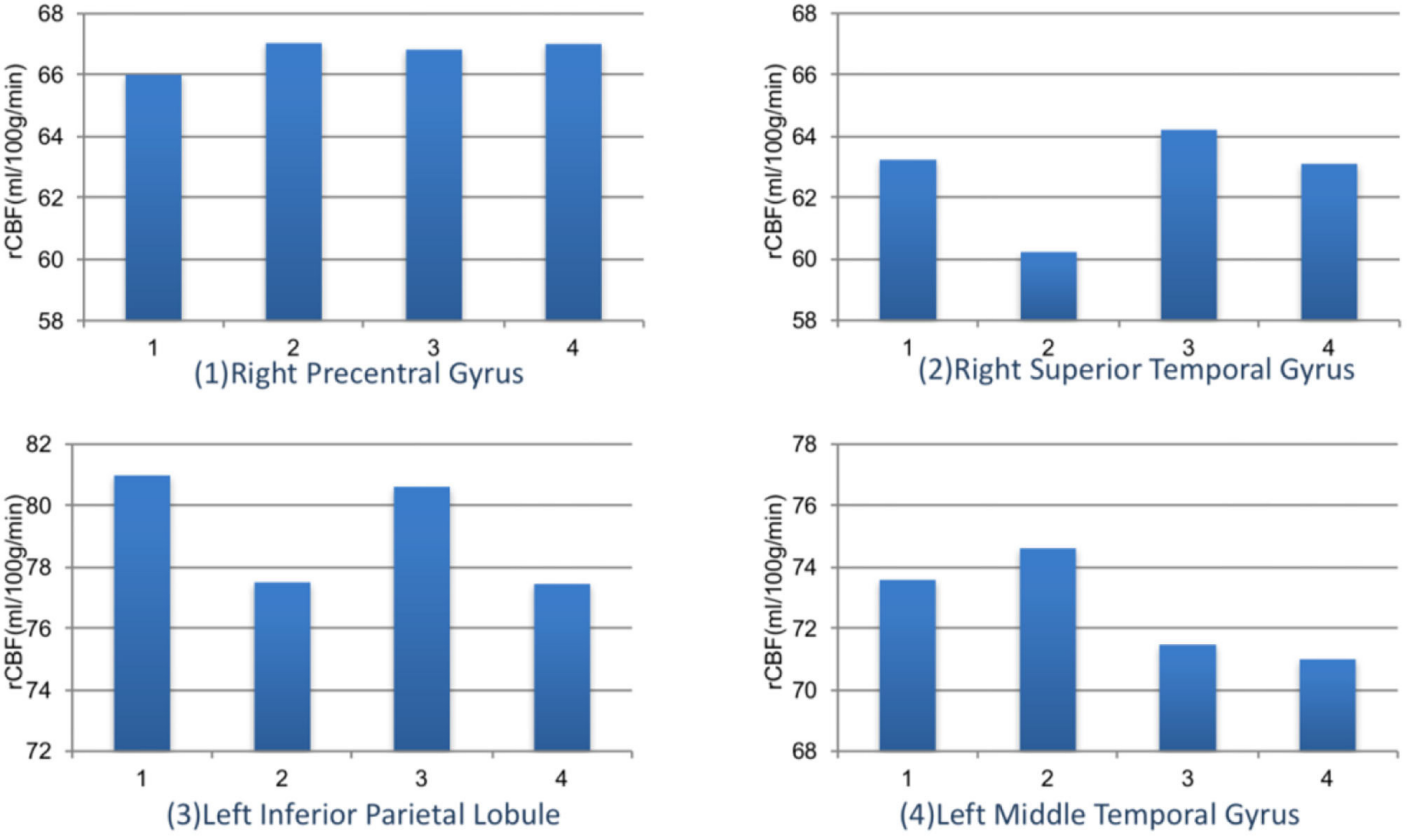
The rCBF signal intensity (ml/100 g/min) changes in four ROIs among different refractive groups. Group 1: emmetropia, Group 2: low myopia, Group 3: moderate myopia, and Group 4: high myopia.

We further compared the magnitude of changes of rCBF in four ROIs among subjects with different myopia degrees (Figure 5). The rCBF changes in the right superior temporal gyrus area for subjects in four groups (emmetropia, low myopia, moderate myopia, and high myopia group) were 63.24, 60.23, 64.20, and 63.11 ml/100 g/min, the increase was the least significant in the low myopia group. In the left inferior parietal lobule, the increase was less significant in the low and high myopia groups, and the mean values for the four groups were 80.99, 77.51, 80.62, and 77.46 ml/100 g/min. One-way ANOVA among groups showed no significant statistical difference (*P* > 0.05).

**Figure 6:**
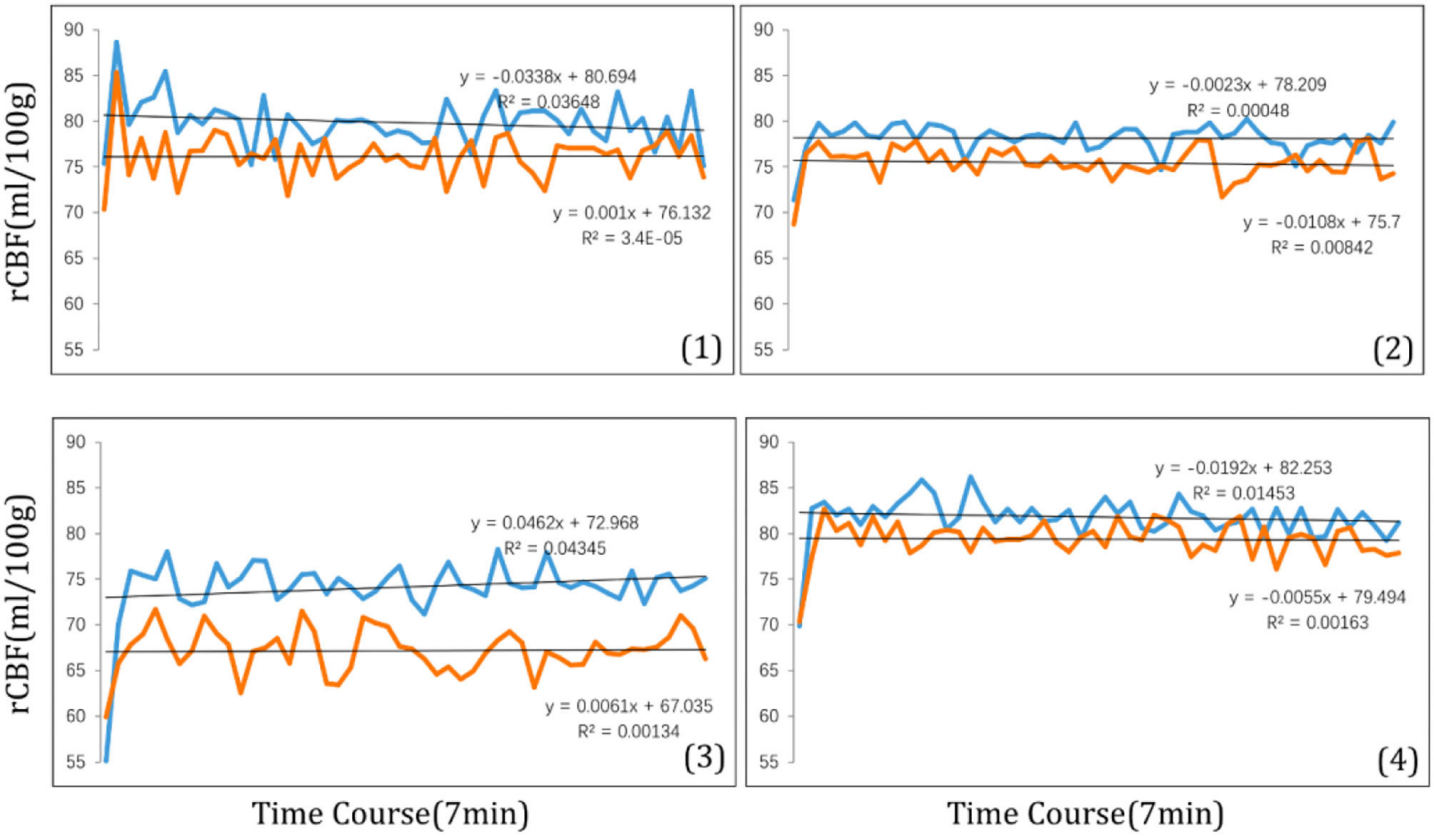
The time course of rCBF signal intensity (ml/100 g) within 7 min in four ROI: (1) right precentral gyrus; (2) right superior temporal; (3) left inferior parietal lobule; (4) left middle temporal gyrus. The blue curve stands for myopic defocus, orange curve stands for clear focus.

The magnitude of the rCBF signal collected at each measurement time point was exported at 8 s intervals. During the 7-min visual tasks, the average rCBF signal intensities in four ROIs were collected (Figure 6) revealing that compared with clear focus visual tasks, average rCBF signal intensity was higher when receiving myopic defocus (*P* < 0.05). However, the changes in signal intensity reached a plateau within 1 min after exposure of defocus.

**Figure 7:**
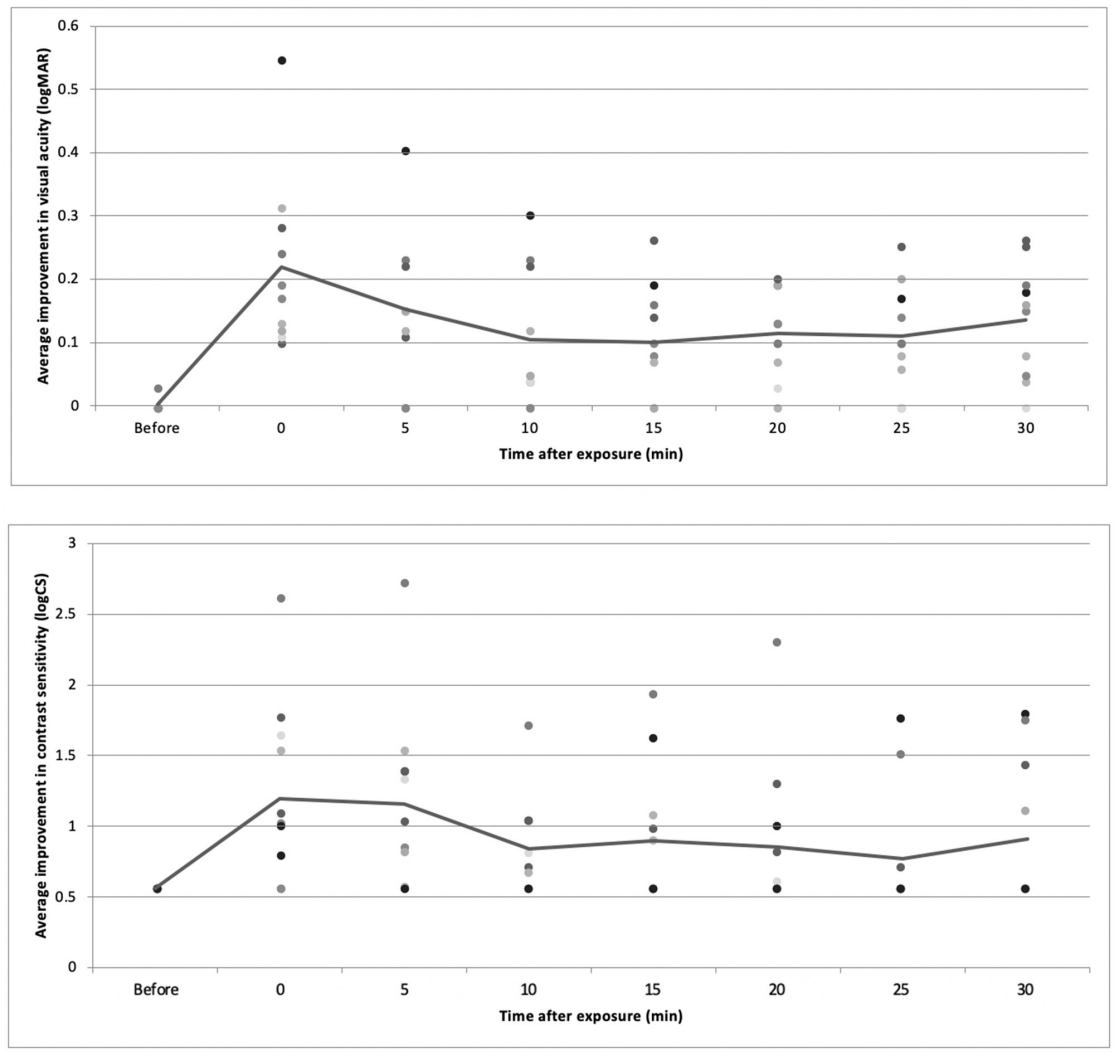
The average change of VA and CS over time.

The logMAR VA and CS were assessed before and after myopic defocus. There were significant decreases of both VA and CS for all subjects at the point of perceiving +2D myopic defocus. Afterward, the defocus visual function was collected at 5 min intervals. The average VA and CS were improved over time (*P* < 0.01). Overall, 30 min of defocused viewing produced significant improvement of VA by 0.14 +0.09 logMAR and CS by 0.91 +0.51 logCS, respectively (Figure 7).

## Discussion

With the ASL technique of event-related-fMRI, we found that the myopic defocus visual stimulation induced the function and perfusion increase in the following cerebral regions relative to the clear focus stimulation: right precentral gyrus, right superior temporal gyrus, left inferior parietal lobule, and left middle temporal gyrus. The above regions were related to oculomotor response, enhancement of attention and awareness triggered by visual stimulation, contemplating distance, and recognition of the image. In addition, we found that hyperopic defocus had less improvement in the above regions. It suggested that the mechanism of the defocus signal involved the functional change of CNS. The functional changes will occur shortly after receiving the retinal defocus signal.

The event-related-fMRI method provides a new approach to assessing the neural underpinnings of visual perceptual effects induced by the defocus stimulus. The fMRI has millimeter spatial resolution and second-order temporal resolution. Through acquiring changes in MRI signals caused by changes in local blood oxygenation, blood flow, and volume caused by neuronal activity in the brain, the location, intensity, and dynamic changes of different functional activities can be measured without injury. This reveals the CNS mechanisms of audio and visual perception. In this study, the behavioral results and fMRI findings are consistent and correlated. Myopic defocus can improve visual function within 5 min; at the same time, the increase of blood flow in the visual attention area could be observed.

Animal research showed that visual error signals strongly guided ocular growth. Myopic defocus will slow myopia progression, and hyperopic defocus will accelerate myopia progression (Norton and Amedo, 2006; Benaventeperez et al., 2012; Benaventepérez et al., 2014; McFadden et al., 2014). Transecting the optic nerve can affect the occurrence of lens-induced myopia in lens-induced myopia in chicken, but it does not affect form-deprivation myopia (Wildsoet and Wallman, 1995; Dillingham et al., 2013). The experiment results showed an interaction between lens-induced myopia and CNS, and the set point of the emmetropization was recalibrated after the optic nerve was cut off (Wildsoet, 2003). Additionally, ocular structure altered quickly after the eyeball received the defocus signal. The depth of the vitreous chamber and the choroidal thickness had compensatory change when myopic defocus is imposed on the eye of a human. In this compensatory change, the axial length of the eye was slightly shortened, thereby becoming hyperopic (Read et al., 2010; Chakraborty et al., 2012), suggesting eyes can quickly detect and respond to defocus signals (Zhu et al., 2005). Adaptation experiments in humans showed that myopic defocus could improve vision to a certain extent and if the human is continuously exposed to myopic defocus stimulation, their ability to distinguish and identify objects will gradually improve. Even though the impact of spectacle-wearing on myopia progression has been documented in quite a several clinical studies, the under-correction effect on myopia progression remains controversial (Smith, 2013; Li et al., 2015). The effect of myopic defocus signal is stronger than that of hyperopic defocus signal. Recent studies have found that 2-h myopic defocus stimulation per day can inhibit ocular elongation (Nickla et al., 2017). Transitory myopic defocus stimulation can compensate for the myopic effect caused by long-time hyperopic defocus stimulation (Zhu et al., 2003).

Furthermore, it has been discovered that brain structure changes in patients with high myopia due to abnormal sensory input. The study of Guo (Li et al., 2012). found that the density of white matter in the brain was higher in patients with high myopia than in healthy controls, while there was no difference in the density of gray matter. The connection between the visual cortex and the visual connecting region is enhanced under the influence of visual awareness. These pieces of evidence indicated that to better collect and integrate visual signals, the brains of high myopia patients developed an adaptive compensatory change to enhance the functional connection between the visual cortex and the related visual regions. The finding that changes only occur on white matter in the brain suggests that late-onset eye disease affects the structure of the brain in different ways. This is because the volume of gray matter is in a stable state in adulthood, while the development of white matter continues for a lifetime due to its close relation to functional training. The study of Guo (Zhai et al., 2016) further explored the functional connectivity density (FCD) in patients with high myopia, which found that FCD in the left inferior temporal gyrus (ITG), supramarginal gyrus (SMG), and rostrolateral prefrontal cortex (rlPFC) was lower than those of healthy controls. Uncorrected VA is related to the long-range FCD of rlPFC. It is worth noticing that the left ITG is related to the ability of the ventral visual pathway to identify objects. In animal experiments, if the function of the ITG region is damaged, the attention filter function can be compromised (Zhang et al., 2011). The damage of SMG is associated with visual attention deficit (Perry and Zeki, 2000; Corbetta and Shulman, 2002), including spatial and locating attention. This means that the visual attention regulation function of high myopia patients is abnormal. The visual experience affects sensory cortex function because of its plasticity (Kaas et al., 1990; Chino et al., 1992; Gilbert and Wiesel, 1992; Chino, 1995; Lewis and Maurer, 2005). Although the moderate myopic defocuses stimulation may slow the myopia progression, we should note that visual deprivation must be avoided because there are multiple sensitive periods during which experience can influence visual development (Blakemore and Cooper, 1970; Lewis and Maurer, 2005).

The retinal defocus mechanism of myopia has been considered to be mainly regulated locally, while some scholars believed the possibility that the defocus mechanism involves brain regulation. The current study validated the hypothesis: the adaptive change and the improvement of visual function induced by myopic defocus may be associated with functional changes in CNS. The right precentral gyrus locates at Brodmann44 and controls both ocular and somatic movement (Iacoboni et al., 1997). Saccade occurs when the visual stimulation is mainly composed of low or high spatial frequency components (Burr et al., 1994), and uncorrected myopia will lead to increased saccadic eye movement (Ghasia and Shaikh, 2015). The function of ocular movement is to shift the visual attention to fix on features that are of particular interest at the scene, and this function is strongly related to visual attention. The left middle temporal locates at Brodmann21 and is related to distance recognition. The right superior temporal gyrus locates at Brodmann22 and is associated with the ability to identify the object in the ventral visual pathway. In the animal experiment, if this area is damaged, the attention filtering function will be compromised (Zhang et al., 2011). The left inferior parietal lobule locates at Brodmann40 and is also related to visual attention (Goldberg et al., 2002). The attention function network locates in the parietal region and is a feedback source that acts on the “top-down” signal pathway in the visual cortex (Pessoa et al., 2003). The attention function can enhance the perceived sensitivity visual stimulation recognition while reducing the surrounding interference. Attention plays a key role in processing audio and visual stimulation. For example, when we receive a large amount of visual information from the external environment, the irrelevant signal that fails to draw attention will not be processed by the visual system. In addition, from the perspective of perception, this type of signal will not reach the consciousness level (Lavie, 1995). A previous study in an experiment of Reinhart revealed the possibility of improving VA through CNS, which showed that 20 min of transcranial direct-current stimulation (tDCS) to the visual cortex could improve VA by 15%, and the improvement was accompanied by changes in CS at high spatial frequencies (Reinhart et al., 2016). It could be an inspiration for future study targeting the impact of the tDCS on the attention and recognition functional region of the brain. The increasing rCBF caused by myopic defocus indicated a potential way of therapeutic training on myopia.

Through establishing a control group and using the same visual pattern, the brain function signals were compared under the myopic defocus visual stimulation and clear focus visual stimulation. It was found that myopic defocus visual stimulation can lead to more increase in attention and awareness function, suggesting myopic defocus visual stimulation activated stronger advanced central visual processing function. This method excluded the attentional function caused by the visual signal. One of the limitations of this study is that it only proves that the cerebral functional changes when receiving a visual signal, which does not constitute sufficient evidence to confirm that this change of function may cause ocular changes and molecular signal transduction changes. However, several studies have shown that there will be rapid and slight ocular changes and visual function improvement when the eye receives a myopic defocus signal. The future study will focus on ratifying the causal-effect relationship between the ocular changes and brain function changes by further exploring the “top-down” neural pathway in the brain. In addition, the limited number of subjects in each refractive group attributed to the failure to identify whether there are statistically significant differences between the sub-group of refractive error. In the future, we will expand the sample size to further verify whether the difference does exist based on our current exploratory analysis.

## Conclusion

With the ASL technique of event-related-fMRI, the study found that myopic defocus visual stimulation induced the function and perfusion increase in the right precentral gyrus, right superior temporal gyrus, left inferior parietal lobule, and left middle temporal gyrus. The abovementioned cerebral regions were related to oculomotor response, attention and awareness, contemplating distance, and recognition of the image. The study on the relationship between CNS and myopia will help us understand the changes in CNS induced by refractive correction, suggesting the potential role of adult brain function training and brain plasticity in the mechanism of myopic defocus.

## Data Availability Statement

The raw data supporting the conclusions of this article will be made available by the authors, without undue reservation.

## Ethics Statement

The studies involving human participants were reviewed and approved by Institutional Review Board of Beijing MRI Center for Brain Research. The patients/participants provided their written informed consent to participate in this study.

## Author Contributions

NW and S-ML contributed to acquisition of funding, collection of data, and general supervision of the research group. BW contributed to design, data analysis, and revision of the manuscript. M-TK contributed to design, collection of data, analysis of results, and drafting of the manuscript. A-RR contributed to design, data analysis, and drafting of the manuscript. MY helped to the manuscript revision. JG, XL, and JD contributed to the collection of data. All authors contributed to the article and approved the submitted version.

## Conflict of Interest

The authors declare that the research was conducted in the absence of any commercial or financial relationships that could be construed as a potential conflict of interest. The handling editor declared a shared affiliation with several of the authors M-TK, JG, MY, XL, S-ML, and NW at time of review.

## Publisher's Note

All claims expressed in this article are solely those of the authors and do not necessarily represent those of their affiliated organizations, or those of the publisher, the editors and the reviewers. Any product that may be evaluated in this article, or claim that may be made by its manufacturer, is not guaranteed or endorsed by the publisher.
